# Psychological distress in elderly people is associated with diet, wellbeing, health status, social support and physical functioning- a HUNT3 study

**DOI:** 10.1186/s12877-018-0891-3

**Published:** 2018-09-04

**Authors:** Kjersti Grønning, Geir A. Espnes, Camilla Nguyen, Ana Maria Ferreira Rodrigues, Maria Joao Gregorio, Rute Sousa, Helena Canhão, Beate André

**Affiliations:** 1 Department of Public Health and Nursing, Center for Health Promotion Research, Norwegian University of Science and Technology (NTNU), Postbox 8905, 7491 Trondheim, Norway; 2 CEDOC, EpiDoC Unit, NOVA Medical School, Universidade Nova de Lisboa, Lisbon, Portugal; 3 EpiSaude Association, Evora, Portugal

**Keywords:** Mental health, Life style, Diet, Older adults

## Abstract

**Background:**

The increasing proportion of people growing old, demands expanded knowledge of how people can experience successful aging. Having a good life while growing old is dependent on several factors such as nutrition, physical health, the ability to perform activities of daily living, lifestyle and psychological health. Furthermore, unhealthy food intake is found to be a modifiable risk factor for depression in elderly people. To promote elderly’s health and wellbeing, the influence of nutrition, lifestyle, physical functioning, and social support on psychological distress needs exploring. Therefore, the purpose of this present study is to investigate the associations between psychological distress and diet patterns when adjusting for other life style behaviors, wellbeing, health status, physical functioning and social support in elderly people.

**Methods:**

The present study is cross sectional, using data from wave three of the Nord-Trøndelag Health Study (2006–2008). Data include psychological distress measured by the Hospital Anxiety and Depression Scale (HADS), sociodemographic information, measurements of lifestyle behaviours (including diet patterns), wellbeing, health status, social support and physical functioning.

**Results:**

The sample consisted of 11,621 participants, 65 years or older. Cluster analysis categorized the participants in two food clusters based on similarities in food consumption (healthy *N* = 9128, unhealthy *N* = 2493). Stepwise multivariable linear regression analyses revealed that lesser psychological distress in the elderly was dependent on gender, diet, smoking, better scores on health and wellbeing, social support and less problems performing instrumental activities of daily living.

**Conclusion:**

Knowledge about the influence of diet patterns in relation to psychological distress provide valuable insights into how society can promote healthy lifestyles to an ageing population, e.g. by increasing older people’s food knowledge.

## Background

The proportion of people older than 65 years is growing faster than any other age group [1]. Recognition of this require expanded knowledge on how this large portion of population can experience successful ageing. Successful ageing is about staying healthy [2] and connected to a diversity of factors, such as psychological resources, life satisfaction, social participation, functioning and personal growth [3]. Wellbeing is also linked to health and age and refers to how people experience the quality of their lives [4], comprising emotional responses, feelings of happiness, sadness, anger, stress, purpose and meaning in life [5]. The process of successful ageing [1] is about extending healthy life expectancy and quality of life for all people as they age, but it is also about optimizing opportunities for health and participation in social, economic, cultural, spiritual and civil matters. Lack of social support has shown to have a negative influence on future quality of life among elderly people [6].

As people grow older, the prevalence of chronic illness is increasing, which challenge the health system to make easily accessible services to meet the growing needs for chronic illness management, risk reduction, promoting healthy lifestyles, and improving the aging population’s quality of life [7]. Wellbeing have a protective role in health maintenance for older people [8], where nutrition and diet prevent frailty. Frailty is a continuum of being at risk of losing, or having lost, social and general resources, activities, or abilities that are important for fulfilling one or more basic social needs during the life span [9]. Also, sociodemographic factors, lifestyle, functional impairments [10], higher level of depression, poor self-rated health and negative affect [11] are shown to be significantly associated with frailty. One study found a strong association between depression and incident of frailty in older women [12].

In summary, a 2017 review [11] states that a wide variety of factors, such as sociodemographic, physical, biological, lifestyle, diet, activities of daily living (ADL), and psychological factors (e.g. depressive symptoms) are associated with frailty. A healthy diet includes food types like fruits, vegetables, fish, whole-grain bread and water [13]. Thus, healthy diets also consist of food variety, enabling food components to be met adequately and comprehensively [14]. Several publications have revealed that healthy diets are associated with better quality of life [13, 15, 16]. For instance, studies have shown that individuals with a higher score for adherence to a Mediterranean-style diet [17, 18] and higher consumption of low-fat milk and yogurt [19] have lower odds of developing frailty, while a variety and quality of diets in older people [20] is increased by factors, such as social resources, money and help from friends and neighbors.

To improve quality of life of older persons, it is important to screen and develop interventions targeting frailty components [6]. Moreover, as elderly’s psychological status and lifestyle behaviours are associated with frailty, it is important to investigate the influence of life style behaviors and health status variables in relation to psychological distress, in addition to less studied factors such as type of neighborhoods [11]. Therefore, the aim of this study is to investigate the associations between diet patterns and psychological distress in elderly people when adjusting for other life style behaviors, wellbeing, health status, physical functioning and social support by using data from a longitudinal epidemiological observational study in Norway [21].

## Methods

### Sample

The Nord-Trøndelag Health Study (HUNT Study) is one of the largest health studies ever performed, collecting data in four waves, HUNT 1 (1984–1986), HUNT 2 (1995–1997), HUNT 3 (2006–2008) and HUNT 4 (ongoing) [22]. This present study is based on data collected in the HUNT 3 wave (2006–2008). The HUNT3 wave (50,807 participants) contain information on the inhabitants’ sociodemographic status, living conditions, life-styles behaviors and health status. In this study, elderly inhabitants are defined as being 65 years or older in 2006 when the HUNT3 data collection started.

### Variables and measures

The outcome variable was psychological distress measured by the Hospital Anxiety and Depression Scale (HADS) [23]. HADS has been translated into Norwegian and is a valid and reliable measure [24] to assess psychological distress. HADS total score range from zero (best) to 42 (worst) as a continuous variable.

The independent variables consist of sociodemographic variables, variables assessing life style behaviors (diet, smoking, alcohol), social support, overall health, wellbeing, and instrumental activities of daily living (1-ADL). Sociodemographic information contained information on sex (male/female) and living situation (cohabitating yes or no). Variables assessing life style behaviors included diet (healthy or unhealthy diet pattern group), current smoking (yes or no) and alcohol consumption (4–7 times a week, 2–3 times a week, about once a week, 2–3 times a month, about once a month, a few times a year, not at all the last year, and never drank alcohol). Food consumption was collected by asking how often of the following foodstuff the participants normally eat. Then, the diet pattern variable was created by re-coding categorical food variables into continuous variables and calculating the average weekly consumption. A healthy diet pattern was characterized as more consumption of fruits, vegetables, boiled potatoes, oily fish, whole-grain bread and water, while the unhealthy diet pattern consisted of larger amount of/consumption of chocolate/candy, pasta, sausages, sugar free and sugary soft drinks, and whole milk, juice, white and semi-grain bread.

Variables assessing social support was assessed by two items from the Social Cohesion and Support Index (SCS) [25], *«do you have friends that can help you when you need them” and “do you have friends that you can speak to confidentially”,* in addition to one item from the Short Form-8 (SF-8) health survey [26]. The SF-8 question “*to what extent has your physical health or emotional problems limited you in your usual socializing with family or friends during the last 4 weeks”* has the following response alternatives; not at all, very little, somewhat, much or was not able to socialize where lower score indicates less problems. As people’s community is relevant for social support, we included three items (network united, network distrust and network welfare) from the HUNT instrument, Local community [22]. The questions were “*I feel a strong sense of community with the people who live here”* (network united), “*we do not trust each other here”* (network distrust) and *“people like living here”* (network welfare), with answers strongly agree, somewhat agree, unsure, somewhat disagree or strongly disagree.

Wellbeing was assessed by a single item *“thinking about your life at the moment, would you say that you by and large are satisfied with life, or are you mostly dissatisfied, which *is found to be a sufficient and valid measure [27]. The response alternatives were very satisfied, satisfied, somewhat satisfied, neither satisfied nor dissatisfied, somewhat dissatisfied, dissatisfied, very dissatisfied. Overall health was measured with the question *“how is your health at the moment”* with the response alternatives poor, not so good, good or very good. We assessed physical functioning by instrumental activities of daily living (I-ADL), defined as problems performing one or more instrumental activities. The activities were preparing hot food, doing light housework, doing heavier housework, washing clothes, paying bills, taking medicine, being able to move outside the house, go shopping and taking a bus without help [28].

### Analyses

The data was analyzed using IBM SPSS Statistics (version 22) [29]. To investigate differences between elderly dependent on diet clusters, we conducted bi-variate analyses where continuous and categorical variables were analyzed with independent t-tests and Pearson’s chi-squared test, respectively. To investigate independent associations between diet patterns and psychological distress when adjusting for other life style behaviors (smoking and alcohol consumption), wellbeing, health status, physical functioning and social support, we created a regression model where groups of variables were added to the model in five steps (Table 2). After each step, we excluded the non-significant variables. Multiple regression analysis are powerful statistical procedures suited to estimate the linear relationship between an outcome variable and one or more predictor variables [30]. Other principal assumptions in addition to linearity that justify the use of linear regression models are statistical independence of the errors, homoscedasticity and normality of the error distribution. In this study, the assumptions of linear regression analyses, values of inter-correlations, the Durbin-Watson and Variance Inflation Factor were not violated. Only cases with valid values for all variables are included in the analyses. In multivariable regression analyses, the standardized beta coefficient (Beta) compares the strength of the association between the independent and dependent variable when adjusting for other independent variables in the model. The level of significance was set to *p* < 0.05. Only cases with valid values for all variables are included in the analyses. Contribution of the independent variables in the model is expressed as explained variance (adjusted R^2^).

The diet pattern variable was created through K-means cluster analysis [31], which is suitable for data with large sample sizes. Cluster analysis classify individuals with similar traits into separate groups. In this study, we re-coded categorical food variables into continuous variables by calculating the average weekly consumption. Then, we standardized these continuous food variables before running the K-means cluster analysis to make sure that food groups did not influence the clusters with specific frequency [31]. The result from the K-means cluster analyses resulted in two different diet patterns (healthy and unhealthy) which are already published [13]. We created the variable cohabitating by recoding elderly’s living situation into a dummy variable. Those registered as married, or living with a registered partner was given value one and those registered as single, separated, divorced, surviving partner, or widower represented the reference category, value zero.

## Results

The sample consisted of 11,621 participants, 65 years or older in 2006. The sample’s characteristics are presented in Table 1, showing that the healthy diet group consisted of more females, more elderly living with someone, less smokers and less alcohol consumption. In addition, participants in the healthy diet pattern group had a lower level of psychological distress, higher scores on wellbeing, better scores on social support and less problems performing I-ADL’s except from being able to do the shopping without help from others. The internal consistency (Cronbach’s Alpha) of the HADS in this sample was 0.82.

**Table 1:** Descriptive characteristics of elderly dependent on diet pattern group

Variables	Healthy diet cluster	Unhealthy diet cluster	*P*-value
	*N* = 9128	*N* = 2493	
	N (%) or mean (SD)	N (%) or mean (SD)	
HADS total score (0–42) ↓	7.56 (5.1)	8.75 (5.5)	.000*
Sociodemographic information			
Female	5119 (56.1)	1181 (47.4)	.000*
Cohabitating	5872 (64.3)	1248 (50.1)	.000*
Lifestyle style behaviors			
Ex-smoker	7282 (83.7)	1871 (83.1)	.006*
Alcohol consumption (1–8) ↓	4.82 (1.9)	5.12 (1.9)	.000*
Overall health and wellbeing			
Life-satisfaction (0–7) ↓	2.25 (1.0)	2.41 (1.0)	.000*
Health at the moment (0–4) ↑	2.66 (0.6)	2.56 (0.6)	.000*
Social support			
SCS (friends support)	7377 (92.2)	1895 (90.5)	.000*
SCS (friends talk)	7204 (90.6)	1875 (89.2)	.059
SF-8 (0–5) ↓	1.54 (0.9)	1.69 (1.0)	.000*
Network united (1–5) ↓	1.78 (0.9)	1.87 (1.0)	.000*
Network distrust (1–5) ↑	3.61 (1.4)	3.36 (1.4)	.000*
Network welfare (1–5) ↓	1.38 (0.7)	1.46 (0.8)	.000*
I-ADL			
Can prepare warm meals without help from others (yes = 1)	5063 (96.1)	1449 (94.8)	.028*
Can do the shopping without help from others (yes = 1)	5063 (95.5)	1453 (94.7)	.196
Can go out without help from others (yes = 1)	5187 (97.8)	1478 (96.7)	.009*
Can do light housework without help from others (yes = 1)	5168 (98.0)	1466 (96.3)	.000*
Can do heavier housework without help from others (yes = 1)	4423 (84.3)	1177 (77.8)	.000*
Can do the laundry without help from others (yes = 1)	4764 (91.5)	1357 (89.9)	.048*
Can pay bills without help from others (yes = 1)	5073 (95.6)	1432 (93.7)	.001*
Can take medicines without help from others (yes = 1)	5097 (98.5)	1453 (97.5)	.009*
Can take the bus without help from others (yes = 1)	4672 (91.4)	1258 (86.7)	.000*

↑Higher score is better, ↓lower score is better

*HADS* = Hospital Anxiety and Depression Scale, *SCS* = Social Cohesion and Support Index, *SF-8* = the Short Form-8 Health Survey, *I-ADL* = instrumental activities of daily living

*p-value < .05

Table 2 presents the results from the multivariable regression analyses. In step 1, both demographic variables, being male and living with someone were associated with less psychological distress. When adding the variable healthy diet pattern to the model, the analyses showed that there was an independent association between having a healthy diet pattern and less psychological distress. In step three of the analyses, other lifestyle behaviors like alcohol consumption and smoking were added to the model. The results showed that increased alcohol consumption and smoking were associated with higher psychological distress. In step four, the analyses still showed that having a healthy diet pattern were associated with less psychological distress when adjusting for demographics, other lifestyle behaviors and instrumental activities of daily living. Instrumental activities of daily living as being able to prepare warm meals, go out, do heavier housework do the laundry and pay bills without help from others were associated with  lower psychological distress. In the final step of the analyses, we added variables assessing wellbeing, health status and social support to the model. The multivariable regression analyses showed that the healthy diet variable still had an independent statistical significant influence on psychological distress when adjusting for the other variables in the model.

**Table 2:** Explained variance in psychological distress among elderly > 65 years old

Independent variables	Psychological distress (HADS)									
	STEP 1		STEP 2		STEP 3		STEP 4		STEP 5	
	Beta	*P*-value	Beta	*P*-value	Beta	*P*-value	Beta	*P*-value	Beta	*P*-value
Sociodemographic										
Cohabitating (yes = 1)	−.029	.008*	−.015	.181						
Sex (male = 1)	−.072	.000*	−.083	.000*	−.078	.000*	−.096	.000*	−.081	.000*
Lifestyle style behaviors										
Healthy diet cluster (yes = 1)			−.097	.000*	−.089	.000*	−.075	.000*	−.048	.000*
Smoker					.057	.000*	.073	.000*	.048	.000*
Alcohol consumption (1–8)↓					.044	.000*	.004	.771		
I-ADL										
Prepare warm meals							−.063	.001*	−.056	.000*
Go out							−.066	.000*	−.025	.080
Do heavier housework							−.076	.000*	.039	.010*
Do the laundry							−.035	.040*	−.023	.148
Pay bills							−.025	.155		
Take medicines							−.003	.850		
Do the shopping							.024	.249		
Do light housework							.030	.088		
Take the bus							−.034	.057		
Health and wellbeing										
Life-satisfaction (0–7) ↓									.297	.000*
Health at the moment (0–4) ↑									-,119	.000*
Social support										
SF-8 (0–5) ↓									.101	.000*
Network distrust (1–5) ↑									−.086	.000*
Network welfare (1–5) ↓									.099	.000*
Network united (1–5) ↓									−.001	.928
SCS (friends talk) (yes = 1)									−.036	.013*
SCS (friends support) (yes = 1)									−.008	.570
Adjusted R^2^	0.07%		0.16%		0.19%		4.8%		24.3%	

Multiple linear regression analyses, standardized coefficients = Beta, * *p*-value < .05

↑ Higher score is better, ↓lower score is better

*HADS* = Hospital Anxiety and Depression Scale, *SCS* = Social Cohesion and Support Index, *SF-8* = the Short Form-8 Health Survey, *I-ADL* = instrumental activities of daily living (Can do the following tasks without help from others)

Moreover, the final model showed that in addition to a healthy diet, being male, being able to prepare warm meals or do heavier housework without help from others, reporting better health and life satisfaction were positively associated with less psychological distress. In addition, more social support in terms of having friends that they could speak to confidentially, experiencing less problems that limited usual socializing with family or friends, and better scores on the community feelings welfare and distrust were all independently statistically significantly associated with less psychological distress. The model as a whole explained 24.3% of the variance in psychological distress.

## Discussion

The aim of this study was to investigate associations between psychological distress and diet patterns when adjusting for other life style behaviors, wellbeing, health status, physical functioning and social support in elderly people.

### The influence of diet patterns on psychological distress

In a previous HUNT study [13], a significant difference in psychological distress between elderly people with a healthy and unhealthy diet was found, but the study did not adjust for possible confounders. In this study, we performed multiple linear regression analysis and adjusted for other independent variables when studying the association between diet patterns and psychological distress. The regression model showed that having a healthy diet was associated with less psychological distress in the elderly when adjusting for other lifestyle behaviours that in other studies have shown to influence older people’s wellbeing [32] and psychological distress [11].

Furthermore, this study confirm findings from other studies showing that sociodemographic determinants are associated with diet quality [33] and social relationships are linked to dietary behavior [34]. Conklin and colleagues [34] found that being single or widowed was associated with lower scores on vegetable variety, and the associations were enhanced when combined with male gender, living alone or infrequent friend contact. Our bi-variate analyses showed that unhealthy diet patterns were more common among males and elderly with fewer supportive friends. These findings confirmed the results from another study [20] showing that varieties and quality of diets in elderly were influenced by social resources and help from friends and neighbors.

Gender differences in food preferences are shown as essential in several studies [33, 35] indicating that females prefer more vegetables and fruits than males [16]. Our analyses found more females in the healthy diet pattern group, and having a healthy diet was associated with less psychological distress. Yet, the multivariable analyses showed that being female in itself were associated with  level of psychological distress.

As the design of this study is cross-sectional, it is not possible to establish a causal association saying that psychological distress is the “outcome” and diet the “exposure” since the relationship could be the opposite direction. However, another study found associations between depressive symptoms and having a diet containing more meat, and that a “meat dietary pattern” was most common in males [16]. Hence, the inconsistent findings regarding the associations between depressive symptoms, diet and gender, need to be further studied.

Furthermore, it is also evident that other lifestyle behaviors, besides diet, influence elderly people’s psychological health. This study demonstrates that smoking and alcohol consumption are independently associated with higher levels of psychological distress when adjusting for variables assessing wellbeing, health status, physical functioning and social support. As such, this study confirms that several lifestyle behaviors (diet, smoking and alcohol consumption) are important domains to target in relation to promoting elderly people’s health and wellbeing. Smoking is for instance found to be more common in elderly with unhealthy diets [16].

However, it is clear that society needs to be aware of several factors, besides diet and other lifestyle behaviors, when aiming for improving elderly people’s psychological status. As this study showed, the ability to prepare warm meals without help from others predicted less psychological distress as well as the ability to socializing with family or friends. These findings, along with evidence from other studies [34, 36, 37], indicate that being social and in interaction with family or friends is of great importance, both for eating healthy and maintaining good psychological health. For instance, the United States has established a variety of programs to improve elderly’s diet, increase social interaction, and delay loss of independence. Several benefits, such as better health, better nutritional intake and improved social interaction were found from these programs [36].

For society, having knowledge of factors associated with less psychological distress is important to promote elderly’s health and detect risk factors [38]. Several health promotion programs are found effective in supporting elderly’s social participation and self-management [9]. In this study, self-management in terms of maintaining the ability to perform activities of daily living, such as preparing warm meals, were associated with less psychological distress. As such, nutrition and food intake play a significant role for both, physical and psychological health in older age [15]. There is evidence that healthy dietary patterns rich in fruit, vegetables, fish, whole grains and starchy low-fat staple foods are likely to promote healthy ageing, including life expectancy and lesser risk of cardiometabolic diseases [39].

### Strengths and limitations

The strength of this study is the large sample of elderly inhabitants. Data includes a detailed registration of food intake, in addition to valid and widely used measurements assessing lifestyle behaviors, health status, wellbeing, physical and social functioning. A limitation of the study is that the questionnaires are all based on self-report, and it has no objective measures of food intake or biological data. However, the data make it possible to investigate associations between self-reported information on lifestyle behaviours such as diet or food intake, and variables assessing elderly’s psychological distress, health, wellbeing, social support and physical functioning and compare our findings with other studies from different countries. Another limitation in this study is the cross-sectional design. Even though it is possible to detect associations between diet patterns, life style behaviors, wellbeing, health status, physical functioning, social support and psychological distress in this sample of elderly people, it is not possible to establish a causal association between psychological distress and the other variables.

## Conclusion

This study found that elderly inhabitants with a healthy dietary pattern were associated with less psychological distress when adjusting for other life style behaviors, wellbeing, health status, physical functioning and social support. In addition, less psychological distress were independently associated with favorable health behaviors such as non-smoking and lesser alcohol consumption, better health status, higher wellbeing, more social support and less problems performing activities of daily living. Knowledge about the influence of favorable health behaviors, such as eating healthy in relation to psychological distress, provide valuable insights into how society can promote healthy lifestyles to an ageing population. Society should use this knowledge to improve the quality of life of older persons, e.g. by develop interventions that increase older people’s food knowledge and knowledge about how to self-manage and promote one’s health.

## Abbreviations

The Hunt Study: the Nord-Trøndelag Health Study WHO World Health Organization QOL Quality of life ADL Activities of daily living HADS the Hospital Anxiety and Depression Scale SCS Social Cohesion and Support Index SF-8 the Short Form-8 Health Survey
